# A hybrid approach to enhance the lifespan of WSNs in nuclear power plant monitoring system

**DOI:** 10.1038/s41598-022-08075-6

**Published:** 2022-03-14

**Authors:** Md Ershadul Haque, Tanvir Hossain, Mahidur R. Sarker, Manoranjan Paul, Md Samiul Hoque, Salah Uddin, Abdulla Al Suman, Mohamad Hanif Md Saad, Tanvir Ul Huque

**Affiliations:** 1 School of Computing & Mathematics, Charles Sturt University, Bathurst, Australia; 2 Department of Electrical & Electronic Engineering, Feni University, Feni, 3900 Bangladesh; 3 Institute of IR 4.0., University Kebangsaan Malaysia, 43600 UKM Bangi, Malaysia; 4 Macquarie Medical School, Macquarie University, Sydney, Australia; 5 School of Computer Science, Queensland University of Technology, Brisbane, Australia

**Keywords:** Electrical and electronic engineering, Power stations

## Abstract

In recent years, the nuclear power plant has received huge attention as it generates vast amounts of power at a lower cost. However, its creation of radioactive wastes is a major environmental concern. Therefore, the nuclear power plant requires a reliable and uninterrupted monitoring system as an essential part of it. Monitoring a nuclear power plant using wireless sensor networks is a convenient and popular practice now. This paper proposes a hybrid approach for monitoring wireless sensor networks in the context of a nuclear power plant in Bangladesh. Our hybrid approach enhances the lifespan of wireless sensor networks reducing power consumption and offering better connectivity of sensors. To do so, it uses both the topology maintenance and topology construction algorithms. We found that the HGETRecRot topology maintenance algorithm enhances the network lifetime compared to other algorithms. This algorithm increases the communication and sensing coverage area but decreases the network performance. We also propose a prediction model, based on linear regression algorithm, that predicts the best combination of topology maintenance and topology construction algorithms.

## Introduction

The use of wireless communication systems and wireless sensor devices (WSDs) driven by battery power simultaneously makes a self-controlled and self-maintained wireless sensor networks (WSNs) technology. Wireless nodes collect the information from the external environment and transmit it through a reliable route to the base station. Nodes are configured in such a way that they can execute an assigned task and transmit the sensed data or conditions to its control unit. A lot of research work has been investigated to produce energy efficient and reliable WSNs. But, nodes have their own limitations, such as limited aggregated energy, smaller communication ability, and short memory in different environments.

Reliable and real time communication are one of the vital issues to be covered by the entire electrical power systems under continuous monitoring for sustaining uninterrupted power supply to the consumer. The collapse of apparatus, natural disaster, etc., provoke power supply failure that can be eliminated by detecting fault rapidly using the integration of real time communication with the systems. The importance of WSNs technology to be implemented into power systems is drawing notable attention since conventional wired communication systems are very expensive, massive to install, and difficult to maintain regularly^1–4^.

A smart power system is a modern electrical technique which promises to promote efficiency, reliability, and safety of integrated power generation, distribution, transmission, etc., through autonomous control systems and communication systems^5^. WSNs are operated by the limited energy of batteries as mentioned earlier in this section. As a consequence, the actual lifetime of the network relies on the controlled use of battery power. For a large WSNs system or risky situation, the lifetime of it is often intended for more than one year to avoid the change of battery of the nodes more frequently^6^. On the other hand, solar energy powered WSD is an alternative to conventional battery powered and can beget a massive amount of cost for highly dense WSNs. Moreover, during the period of lack of sunlight, the efficiency of the solar energy powered WSDs can be notably decreased which is not a negligible matter for WSNs system^4,7^. A crucial affair in regard to the WSNs for such a situation as a power generation station is the hardware failure of a sensor device since it can disrupt the entire system. A WSNs system detects its defective device as soon as possible and sends respective information to the base station to recover it immediately. Several fault detection and recovery processes of WSDs in a WSNs system are represented in^8–13^. However, a low cost real time communication system has appeared as a significant framework to ensure an efficient and safe power system.

It is often yearned to confirm the relationship between two or more correlated variables to predict a situation. Regression algorithms allow predicting an outcome by mathematically analyzing the relationship between two or more mutually dependent variables. In recent times, the development of these regression algorithms is being advanced as they yield an accurate prediction and easier interpretation of a large data set^14^. The major contributions included in this work are:

- designing the WSN infrastructure for monitoring nuclear power plants through optimized deployment of WSDs.
- set up and run the WSN infrastructure through a hybrid approach of the combination of the topology maintenance (TM) and topology construction (TC) protocols.
- developing a linear regression model to predict the best deployment of TM and TC algorithms together.

The rest portion of this article is organized as follows: “Related work” section provides an overview of related work from the literature. The methodology of this work is represented in “Methods” section. The discussion of the evaluation of performances and related research outcomes are included in “Result analysis and discussion” section. “Conclusion” section presents of the summary of our contribution and the future research directions.

**Figure 1:**
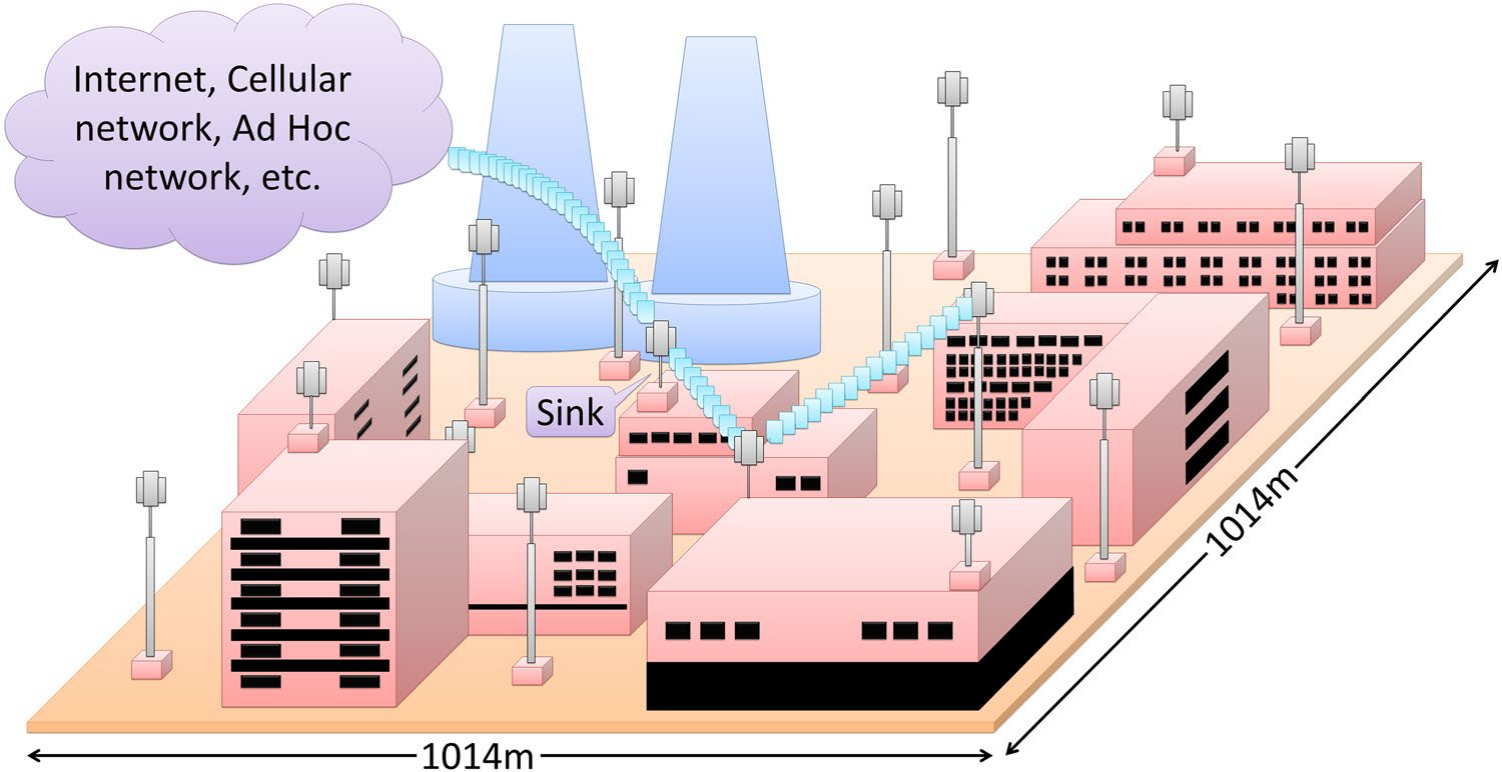
An imagination of WSNs employed nuclear power plant.

## Related work

We divide the related work section into two parts. In the first part, we discuss the research works related to the monitoring systems using the WSNs. And, in the second part, we discuss the relevant regression techniques used in WSNs.

### WSN based monitoring system

Haque et al.^15^ discuss different types of TM algorithms that can be used in the internet of things (IoT) based WSNs, in their paper. Their research economizes on the energy consumption of nodes of the topology over its lifetime to keep up the maximum link within the entire topology. The WSN has a versatile application area, such as the power stations, the health care systems, and structural health monitoring.

The WSN system is one of the most popular technologies used in an electrical power station for monitoring purposes. Sarobin et al.^16^ present a WSN system for managing a smart power station, specifically, the wind farm, in their paper. In their work, the heterogeneous nodes are distributed in the desired region to ensure total connectivity. Abdulwahid et al.^17^ discuss the effects of WSNs in various power system fields, namely substation, control room, and transformer room. They estimated the packet delivery ratio, communication delay, and energy consumption of a WSN, consisting of fifteen nodes in the area of 50 × 50 m^2^. Another study presents that, in the medium and high-density networks, 140 and 1000 nodes are used to evaluate the experimental and simulation results, respectively^18,19^.

Al-Shargabi et al.^20^ present a WSN based health care system. They deployed the nodes in a rectangular area of 1000 meters square. They estimated the potency of different topologies using different numbers of nodes regarding power consumption and packet delivery ratio. They measured various communication interim time cycles between two events at a five minutes cycle, the temperature of a patient room at a one-hour cycle.

In WSNs, real-time and reliable link quality prediction (LQP) are two significant features that are used to identify which link can transmit the data packets more effectively^21^. The random vector functional link (RVFL) algorithm based on LQP is also used widely to estimate the dynamic stochastic features of link quality, and verification of the predicted result^22^. This algorithm converts the SNR sequence into a time-varying sequence and the stochastic sequence based on the characteristics of the link. Then, a prediction model is evaluated by the RVFL network. This prediction model assists nodes of the WSNs to produce a reliable route among themselves.

Ali et al.^23^ propose a dynamic algorithm named time-based reliable link (TBRL) to measure the environmental condition of a smart city using WSNs. The performance of the TBRL was found better than depth-based routing (DBR) and reliable energy efficient routing (REER) protocol. Besides, TBRL is also useful for machine learning algorithms when detecting underwater materials and classifying them.

### Linear regression techniques

The regression models are widely used in medical applications. Brito et al.^24^ present a data set of body mass index (BMI) and mid-upper arm circumference (MUAC), collected from the hospitalized patients, and derive a dependency between BMI and MUAC. They estimate the linear relationship between these two parameters using the linear regression algorithm. Bielemann et al.^25^ also use the linear regression algorithm to estimate the whole body fat content for both men and women.

In their seminal work, Bebbington et al.^26^ represent an estimation of future trends in demand for hand surgery up to 2030 year using a linear regression algorithm. They also estimate a variation in workload over time by integrating age-specific population data with the regression model. To do their analysis, they collected the statistical data of Dupuytren disease, Carpal tunnel syndrome, Cubital tunnel syndrome, and Trigger finger from different hospitals in 1998–2011.

The regression models are also used in structural health monitoring application. Mottahedi et al.^27^ propose a linear regression model that can predict the outcome of utilization of energy based on the shape of different buildings in two, unlike circumstances, such as cold, dry, and warm marine. They use seven regression models for each of the circumstances and separately observe the influences of variations. The regression models are also used in a structural health monitoring application. Their regression model can also find the primary source of energy consumption in different climates. Table 1 presents a simplified comparison between our proposed technique and other existing research work.

**Table 1:** A summary of the comparison of the proposed approach with others related works.

Reference	TC algorithms	TM algorithms	Distribution kind	Application area
Our work	A3Cov	HGTTRecRot, HGETRecRot, and NoTM	Uniform	Nuclear power plant monitoring
^15^	CDS–Rule–K, A3Cov, A3, and EECDS	DGETRec, SGETRot, HGTTRecRot, ELPDSR, DGTTRec, and SGTTRot	N/A	Structural health monitoring
^28^	A3, and EECDS	SGTTRot, DGETRec, DGTTRec, and HGTTRecRot	Gaussian	Power plant monitoring
^29^	A3, CDS–Rule–K, EECDS, K–neigh, and A3Cov	NoTM	Gaussian	Structural health monitoring

## Methods

The Architecture of the tree based WSNs system continues to involve the new devices over time to enhance its duration of operation and coverage areas. The entire network is comprised of uniformly distributed sensor networks throughout the target area and one sink node that collects all the information and leads communication of WSNs with other networks, such as, internet, cellular network, ad-hoc wireless network, so that it can be monitored and controlled from an external world.

In this section, we discuss the path loss models of the transmitter and receiver of the sensor devices in “Path loss model” section, the energy consumption model of sensors of the WSN in “Energy dissipation model” section, and the coverage sensing model of the sensor in “Sensing model” section. We discuss our proposed technique in “Topology control and maintenance algorithms” section, a specific type of topology construction algorithm that we have used in our proposed technique in “A3 based topology control algorithm” section. Finally, we present the used prediction technique in “Prediction model” section and “Data collection and processing” section.

### Path loss model

The sender device sends the information through a radio channel to the receiver. The receiver device receives the information of the sender device when the power of the signal is more than its transceiver sensitivity. The effective communication between the sender and receiver devices depends on the path loss. The path loss between these two devices depends on the spatial distance between these two devices and the signal frequency^30^.

**Figure 2:**
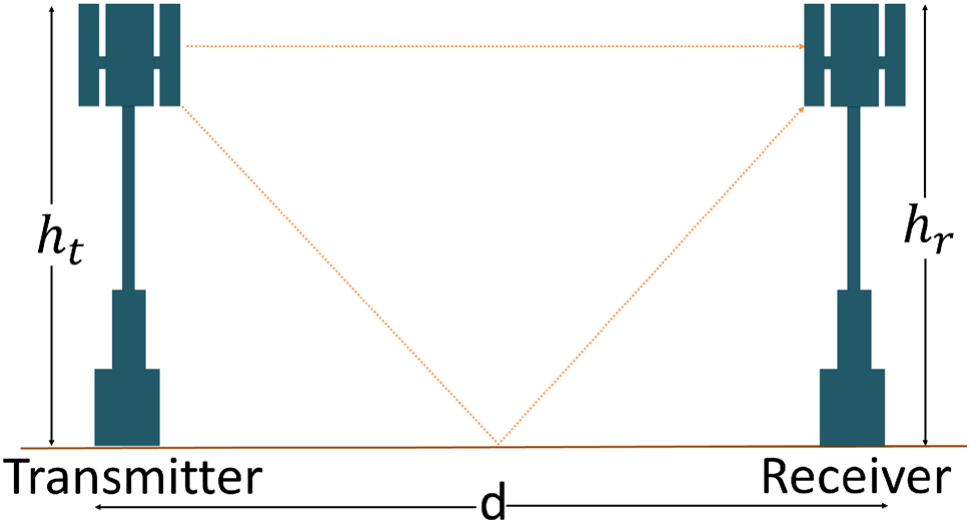
Two ray ground propagation model.

There are different types of path loss models, such as the two ray ground propagation model^31^, Friis’ free space propagation path loss model^32^, and The log distance path model^33^. We use Friis’ free space propagation path loss model when there is no obstacle between sender and receiver devices. The log distance path model is commonly used when we consider numerous obstructions and reflections between sender and receiver devices. The two ray ground propagation model is frequently used compared to the other two path loss models as it combines the benefits of both of these models^34^. We use the two ray ground propagation model in our proposed technique. Figure 2 presents the two ray ground propagation model consisting of a transmitter and a receiver. In Fig. 2, $h_t$ and $h_r$ are the height of the transmitter and receiver antenna, respectively; $G_t$ and $G_r$ are the gain of transmitter and receiver antenna, respectively.

The power of the received signal ($P_r$) at the receiver is defined in Eq. (1)^31^. In Eq. (1), $P_t$ is the power of the transmitted signal at the transmitter device, *d* is the distance between the transmitter and the receiver, and $C_t$ is a constant. The value of $C_t$ depends on the sensitivity of the transceiver. Equation (2) is another formation of Eq. (1). Equation (2) is used estimate the radius (*d*) of the coverage area of the transmitter.

1 $$\begin{aligned} P_r= & {} \frac{P_t.G_t.G_r.{h_t}^2.{h_r}^2}{d^4} = C_t \frac{P_t}{d^4} \end{aligned}$$

2 $$\begin{aligned} d= & {} \root 4 \of {\frac{C_t.P_t}{P_r}} \end{aligned}$$

**Figure 3:**
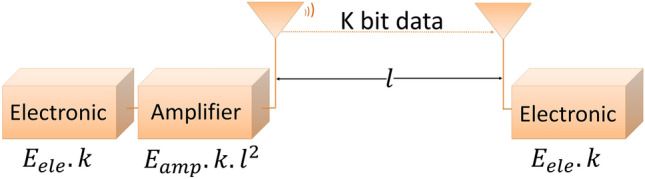
A system in the prospect of first order energy dissipation model.

### Energy dissipation model

We use the energy dissipation model to evaluate the energy consumption of sensor devices in WSNs for using different communication algorithms. The first order radio model of Heinzelman et al.^35^, shown in Fig. 3, is considered as one of the simple and widely used energy dissipation models. According to this model, the spent energy of the transmitter and receiver can be estimated using Eqs. (3) and (4) respectively, considering that a system transmitting *k* bit data at a distance of *l*. In Eq. (3), $E_{tx}$ is spent energy of transmitter device to transfer *k* bit data, in that $E_{ele_{tx}}(k)$ and $E_{amp_{tx}}(k,l)$ are the amount of energy consumed by the transmitter circuit and amplifier respectively. In Eq. (4), $E_{rx}$ is spent energy of receiver to capture *k* bit data. It is identical to the consumed energy $E{ele_{rx}}(k)$ of the receivers circuit.

3 $$\begin{aligned} E_{tx}= & {} E_{ele_{tx}}(k)+E_{amp_{tx}}(k,l) = E_{ele}.k+E_{amp}.k.l^2 \end{aligned}$$

4 $$\begin{aligned} E_{rx}= & {} E_{ele_{rx}}(k) = E_{ele}.k \end{aligned}$$

### Sensing model

A sensing model of sensors is used to estimate the sensing coverage of sensor devices in the WSN^36^. Two widely used sensing models are the binary sensing model and probabilistic sensing model^37^. The binary sensing model labels an event with the highest probability, i.e., 1, when it detects the event within the sensing range. The probabilistic sensing model yields a more accurate description of the sensing detection scheme than binary sensing^38^.

**Figure 4:**
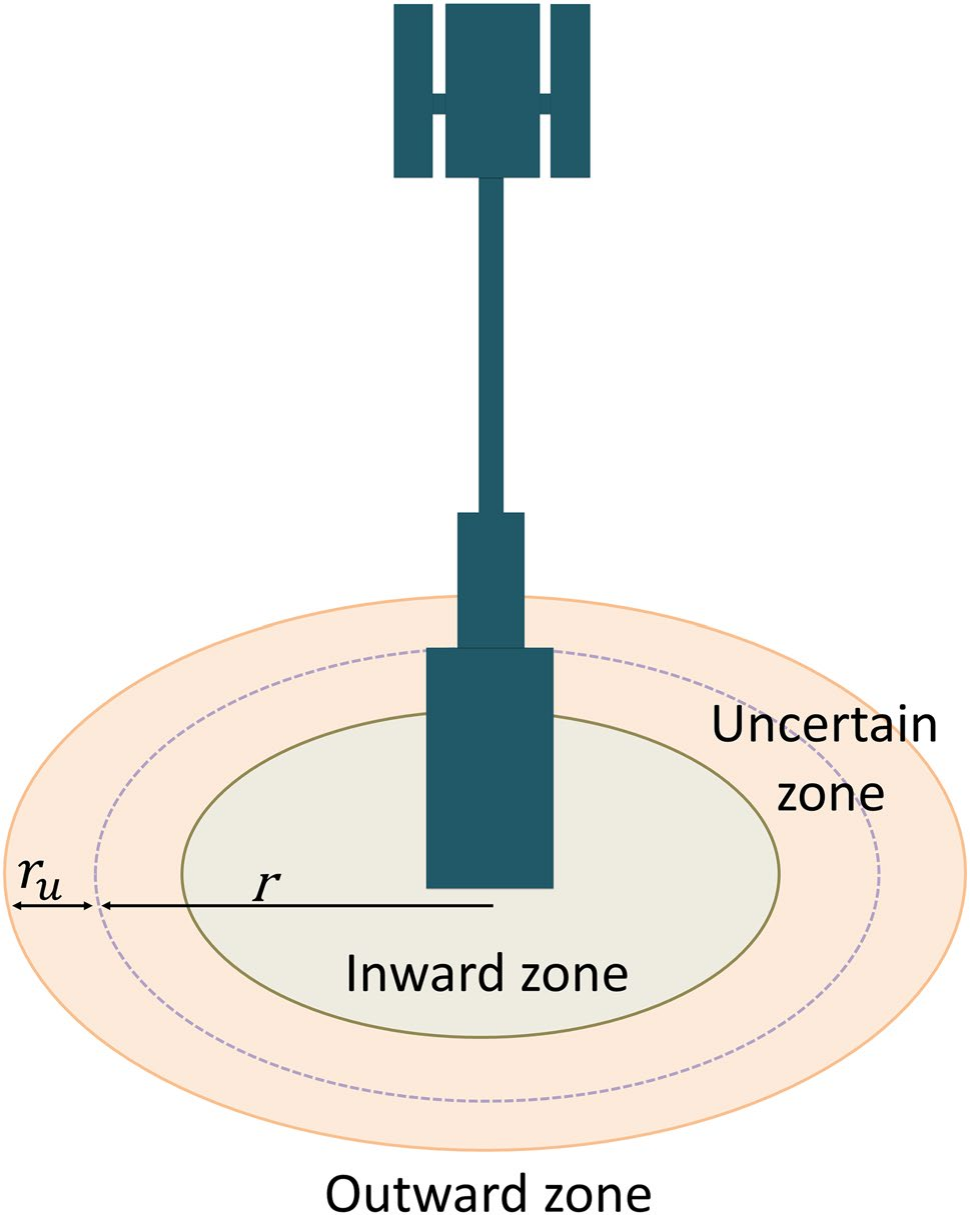
The probabilistic sensing model.

The probabilistic sensing model assumes the range of a sensor is classified into three zones, shown in Fig. 4: inward zone, uncertain zone, and outward zone^39^. In the inward zone, the probability of detecting an event is one. The outward zone has a probability of nearly zero. Probability is exponentially decayed with respect to distance in the uncertain zone. The sensing coverage *C*(*s*) of the probabilistic sensing model conferred by a circular disk of radius *r* is defined in Eq. (5). In Eq. (5), $\alpha =x-(r-r_u)$ and *x* is the euclidean distance between the sensor and event. $\lambda$ and $\beta$ depend on the type of physical sensor devices.

5 $$\begin{aligned} C(s) = {\left\{ \begin{array}{ll} 1,\ for \ r-r_u \ge x \\ e^{-\lambda \alpha ^{\beta }},\ for \ r-r_u<x \le r+r_u\\ 0,\ for \ r+r_u<x \end{array}\right. } \end{aligned}$$

**Figure 5:**
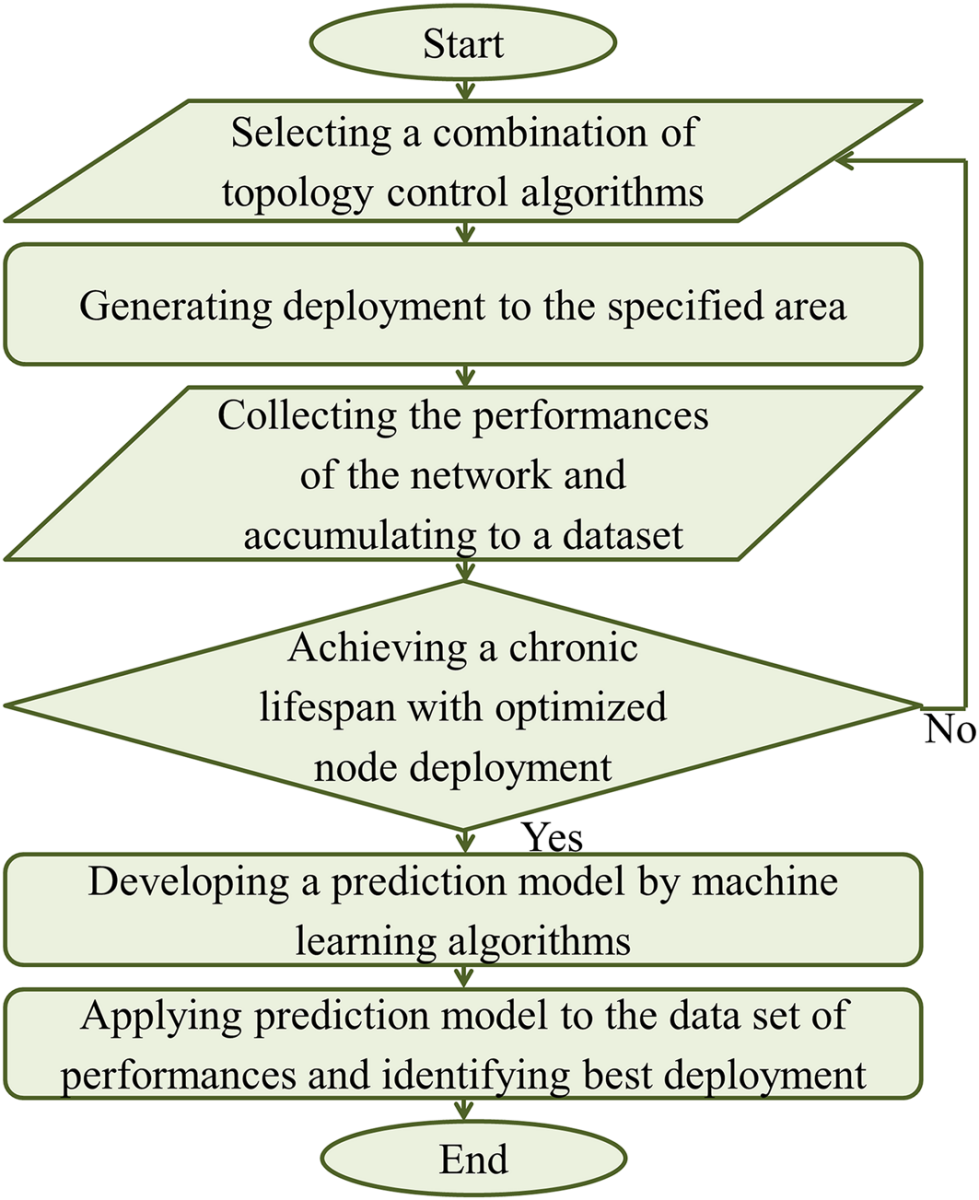
A flowchart of the proposed approach.

### Topology control and maintenance algorithms

A dynamic barrier coverage (DBC) and distributed coverage game algorithm (DCGA) combined model is proposed to produce a complete monitoring environment for a smart power system^22^. DBC and DCGA follow the economical distribution of nodes, adjustment of connectivity, real time calculation of connectivity probability, and the behavior of the deployment area and robots, respectively. A 74,500 × 143,400 m area of coverage was considered for the simulation setup.

The idea of TC, as transacted to promote the lifetime of a WSNs by reducing the energy consumption of the battery driven devices but perpetuating coverage and connectivity, is one of the most significant methods in the feature of WSNs. At the initial, homogeneous sensor nodes build a plane topology to measure various conditions by using maximum energy. Then, the TC mechanism establishes a new reduced topology for a certain period as long as the involved devices remain operative.

The Critical transmission range (CTR) and range assignment (RA) problems are two popular approaches under this concept to control the transmitting power through a central method. CTR is utilized to compose a total connective network calculating the minimum communication range between all kinds of nodes in the network. On the other hand, RA is based on optimal transmitting power of every node but preserving the connectivity of the network. Geometric random graph imparts a higher probable analytic solution of CTR producing connected topology. Considering , *n* number of nodes are distributed uniformly into a square deployment area of edge *l*. In a two dimensional dense network, Penrose et al. estimates the value of CTR by Eq. (6) is given below^40^,

6 $$\begin{aligned} CTR_{dense} = \sqrt{\frac{log(n)+f(n)}{n\pi }} \end{aligned}$$

Here, *f*(*n*) is a dependent function to *n* such that $\lim _{n \rightarrow \infty } f(n)= +\infty$. *log*(*n*) is natural logarithm of *n*(*ln*(*n*)). RA may be a better substitution where computation of CTR is supposed to be a hard and costly operation. Furthermore, CTR is in fact a particular case of RA where the nodes needed the same transmitting power. The RA problem of a set of *n* number node into *d* dimensional area is a function, which produce a strong connective network but optimize the total energy consumption of network given by how much power is consumed by all *n* nodes^41,42^.

WSNs should expect a new reduced topology after ending the activity of subsisting active topology through the combination of previously inactive nodes. TM mechanisms restore, rotate, and recreate the network topology over time to time while required involving the role of nodes as much as possible. Moreover, the global technique tries to commit a global optimal solution taking into consideration all the nodes of a network while the local technique commit a local optimal decision taking into consideration only a few set of nodes. Triggering variables, namely, time based, energy based, random, failure based, density based, and combination of them have significant worth in the network.

The TM mechanism is initiated as soon as the TC method forms the reduced topology. It collects the status of the reduced topology and triggers a new phase of TC mechanism while required. This cycle is continued many time to lead an efficient network having a maximum lifetime. The TC and TM algorithms employed to install a complete topology control method are discussed in *A3 based topology control algorithm* section. The performance of a network in terms of alive nodes, active nodes reachable from sink, ratio of communication coverage, and ratio of sensing coverage with respect to numerous time step is accumulated in a data set. Linear regression algorithms may be a better preference for data analysis and producing a precise estimation of a desirable parameter. A prediction model based on linear regression algorithms is demonstrated in Prediction model section. A flowchart narrating the methodology as well as how it proceed is given in Fig. 5.

### A3Cov based topology control algorithm

**Figure 6:**
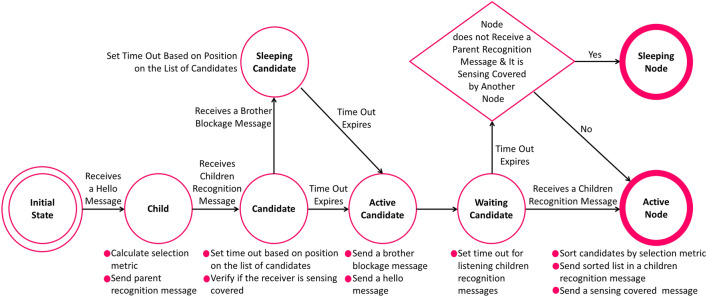
A3Cov algorithm.

In^43^, authors presented an A3 based TC algorithm to estimate full communication coverage. It is an energy-efficient extension of the growing tree mechanism that keeps the network connectivity by turning on necessary nodes while keeping unnecessary nodes into the off mode. The nodes are incorporated by communicating hello messages, parent and children recognition messages, and sleep mode messages to build a tree. The priority of involving a new node in the tree is evaluated by the algorithm using a metric that is proportionally related to the rest energy of node and distance between parent node as given in Eq. (7).

7 $$\begin{aligned} M_{x,y} = W_E \frac{E_x}{E_{max}} + W_D \frac{S}{S^*} \end{aligned}$$

In Eq. (7), $W_E$ and $W_D$ are the weights for the rest of the energy in the node and the distance from the parent node, respectively. $E_x$ is rest energy into the node *x* and $E_{max}$ is maximum initial energy. The strength of the signal received from the parent node is *S*. $S^*$ is the minimum value of *S* that enables communication depending on the sensitivity of the receiver node. A3 Coverage (A3Cov), a grade ahead than the A3 TC algorithm, was proclaimed in^44^. This algorithm is defined in this framework that if all the nodes of a network reside under the sensing coverage of an active node, then the deployment area is covered in the dense network. It works in the process similar to the A3 algorithm. The diverse among these two algorithms is that A3 be defined by the communication range of the active node,whereas A3Cov by the sensing range.

A3Cov accomplishes an improved solution to the coverage problem than the previous version. The operations of the algorithm include neighbour discovering, children selecting, second opportunity, and sensing selecting. A schematic overview of the tasks is shown in Fig. 6. The static TM mechanism begins to work calculating all the probable disjoint topologies in the time of the initial TC process. Then, it stores these topologies into the memory so as to switch out each other according to the requirement. The dynamic TM mechanism works in the sense that it triggers TM mechanisms to calculate a newly built topology when gets a request. The hybrid global TM mechanism can be regarded as a combination of static and dynamic global mechanism triggered based on both energy threshold and time threshold. It calculates all the topologies reduced at the time of processing of initial TC mechanism and queries a new topology once the active topology is unable to carry on.

The routine of no topology maintenance (NoTM) is nothing but the function of initially constructed topology as long as the sink realizes that there is absent of TM mechanism. Hybrid global time based topology recreation and rotation (HGTTRecRot) mechanism rotates the active reduced topology for one of the preplanned topologies at the every interim of every time set. Recreation of that reduced topology is redacted if the sink missed out in connectivity in the existing active topology. Every time, hybrid global energy based topology recreation and rotation (HGETRecRot) triggers the rotation when a node arrives at a critical threshold of energy. In current active topology, when the sink is isolated from the network, the recreation process is performed^45^.

### Prediction model

In our proposed technique, we use the linear regression technique. The linear regression analysis^46^ of a dataset is defined in Eq. (8); where, *x* and *y* variables are two different information sets. Note that a dataset usually consists of two or more sets of information. Equation (8) is so fitted that the sum of the total squared vertical difference between each actual data of the data set and that best fitted line, called residual, is minimized. The pair of two actual statistical *x* and its corresponding *y* value represents a data point of the dataset where *y* is collected according to *x*. Now, pick out a data point *i* from a dataset of *n* data points. At that point, the magnitude of residual $R_i$ is defined in Eq. (9).

8 $$\begin{aligned} Y= & {} \beta _0+\beta _1x \end{aligned}$$

9 $$\begin{aligned} R_i= & {} y_i-Y_i \end{aligned}$$

In Eq. (9), $y_i$ is an actual value and $Y_i$ is the value of prediction for the data point *i*. Note that lowercase *x* and *y* represent the known value of the data set and uppercase *Y* denotes the predicted value. The coefficients $\beta _0$ and $\beta _1$ of the linear regression model called intercept, and slope respectively. If all the value of $R_i$ is zero, the model gives an equation where all the data points abide in the predicting line. Therefore, the residuals need to be minimized to achieve better accuracy of the model.

10 $$\begin{aligned} S = \sum _{i=1}^{n} {R_i}^2= \sum _{i=1}^{n} (y_i-\hat{\beta _0}-\hat{\beta _1}x_i)^2 \end{aligned}$$

The least-square technique, defined in Eq. (10), is the most common method based on the values of coefficients are identified^47^. In Eq. (10), we consider that $\beta _0$ and $\beta _1$ of Eq. (8) are $\hat{\beta _0}$ and $\hat{\beta _1}$, respectively. Partially differentiating *S* with respect to $\hat{\beta _0}$ and $\hat{\beta _1}$ we get Eqs. (11) and (12) respectively. Finally, we can estimate the values of $\hat{\beta _0}$ and $\hat{\beta _1}$, defined in Eqs. (13) and (14) respectively, using Eqs. (11) and (12). In Eq. (14), ${\bar{x}}$ is the mean value of $x_i$ observations and ${\bar{y}}$ is the mean value of $y_i$ observations.

11 $$\begin{aligned}&\frac{\partial S}{\partial \hat{\beta _0}} = -2\sum _{i=1}^{n}(y_i-\hat{\beta _0}-\hat{\beta _1}x_i)=0 \end{aligned}$$

12 $$\begin{aligned}&\frac{\partial S}{\partial \hat{\beta _1}} = -2x_i\sum _{i=1}^{n}(y_i-\hat{\beta _0}-\hat{\beta _1}x_i)=0 \end{aligned}$$

13 $$\begin{aligned}&{\hat{\beta }}_0 = {\bar{y}}-\hat{\beta _1}{\bar{x}} \end{aligned}$$

14 $$\begin{aligned}&{\hat{\beta }}_1 = \frac{\sum _{i=1}^{n}(x_i-{\bar{x}})(y_i-{\bar{y}})}{\sum _{i=1}^{n}(x_i-{\bar{x}})^2} \end{aligned}$$

### Data collection and processing

The dataset for the linear regression analysis is generated by Atarraya simulation package. It contains 456, 1691, and 555 samples of the NoTM, HGETRecRot, and HGTTRecRot circumstances, respectively, of network’s individual four performance metrics, such as the number of alive nodes, the number of active nodes connected to sink, total communication covered area, and the covered area for sensing. This dataset is used as the input of the linear prediction model, discussed in “Prediction model” section. The prediction model was implemented using python 3.0.

The best fitted lines were obtained as shown in “Prediction model’s performance evaluation” section and the validation of the model is determined when the testing variables were fed into the model. The coefficient of determination (CoD) is the most important parameter to interpret the validation of the linear regression model. The CoD ($R^2$), one minus the residual sum of squares divided by the total sum of squares, is defined in Eq. (15)^48^. In Eq. (15), $j_k$, $\hat{j_k}$, and $\bar{j_k}$ stand for the actual value, predicted value, and mean values of observed variable, respectively. Note that the confidence of the predicted lifespan of the WSN, following the prediction model, is increased with the increasing value of CoD.

15 $$\begin{aligned} R^2 = 1-\frac{\sum (j_k-\hat{j_k})^2}{\sum (j_k-{\bar{j}})^2} \end{aligned}$$

Another important parameter, the root mean squared error (RMSE) is also used to interpret the validation of the linear regression model. RMSE is a risk metric that is related to the expected value of the root squared (quadratic) error. The RMSE is defined in Eq. (16); where, $j_k$ and $\hat{j_k}$ correspond to the actual value and predicted value, respectively and *N* is the number of observations. The lower value of RMSE indicates a lesser variation in errors in the predicted lifespan of the WSN.

16 $$\begin{aligned} RMSE = \sqrt{\frac{\sum (j_k-\hat{j_k})^2}{N}} \end{aligned}$$

## Result analysis and discussion

The compatible combination of TM algorithms with TC algorithms is very significant to have the extended lifespan of WSNs. A better performance of WSNs employed in a nuclear power plant using different combinations of TC and TM algorithms is represented in this work. Our proposed approach is simulated on $1014\times 1014\ {\text{m}}^2$ area of the nuclear power plant named “Rooppur nuclear power plant” residing in Bangladesh as imagined in Fig. 1. The evaluated optimum nodes deployment for NoTM, HGTTRecRot, and HGETRecRot are 454, 545, and 558, respectively by distributing the nodes uniformly in the specified area. The parameters of the network are given in Table 2. Three inspections are considered to investigate the lifetime of the network. Firstly, finding the best performances by applying the hybrid TM algorithms with TC algorithms. Secondly, comparing the performance of NoTM with the hybrid TM with TC algorithm in terms of enhancing the lifespan of the network. Finally, assessing the effect on the entire network’s performance of the triggering criteria of the maintenance algorithms (time-based triggering and energy-based triggering). However, small changes in the time and energy thresholds of the triggering criteria should not provide better or worse performances notably. We considered the following performance metrics with respect to time steps.

- The number of alive nodes which indicates the utilization of nodes.
- The number of active nodes reachable to sink, that is, the number of node linked to the sink.
- The covered communication area in terms of the ratio of the covered communication area with respect to the target area.
- The covered sensing area in terms of the ratio of the covered sensing area with respect to the target area.

**Table 2:** Values of the parameters of the network.

Parameters	Values		
	NoTM	HGTTRecRot	HGETRecRot
Number of nodes	454	545	558
Communication radius (per node)	100 m	100 m	100 m
Sensing radius (per node)	20 m	20 m	20 m
Area of deployment	$1014 \, {\text{m}}\times 1014 \, {\text{m}}$	$1014 \, {\text{m}}\times 1014 \, {\text{m}}$	$1014 \, {\text{m}}\times 1014 \, {\text{m}}$
Sink node location	Central position	Central position	Central position
Energy consumption	1000 mJ	1000 mJ	1000 mJ
$E_{ele}$	50 nJ/bit	50 nJ/bit	50 nJ/bit
$E_{amp}$	$10 \, {\text{pJ/bit/m}}^2$	$10 \, {\text{pJ/bit/m}}^2$	$10 \, {\text{pJ/bit/m}}^2$

### Actual performance evaluation

To determine the most significant performance of the optimized network, the considered parameters are given as follow.

#### Number of alive nodes

**Figure 7:**
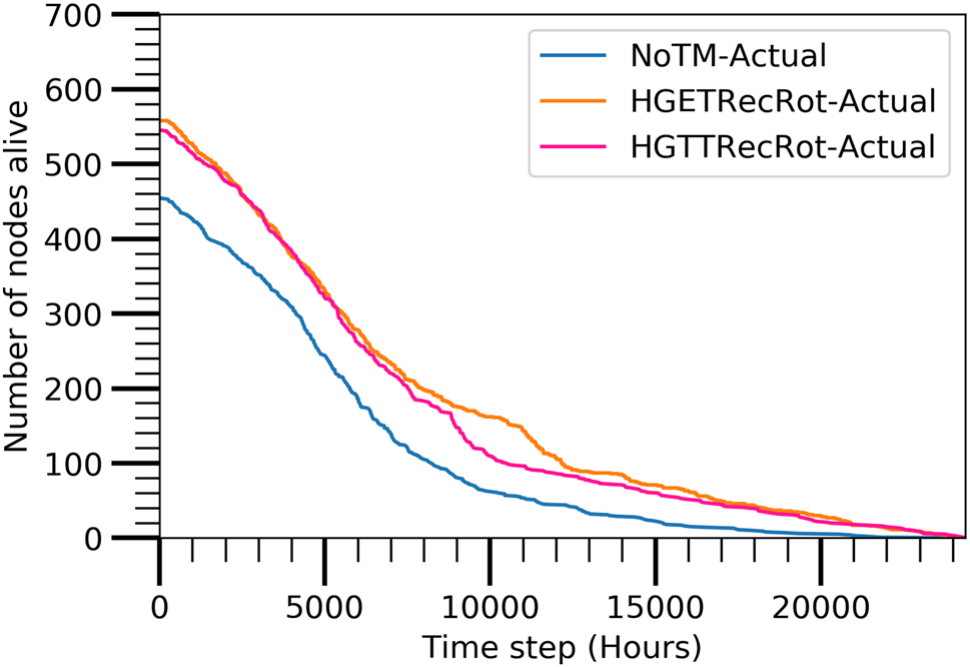
Actual evaluated alive node.

**Figure 8:**
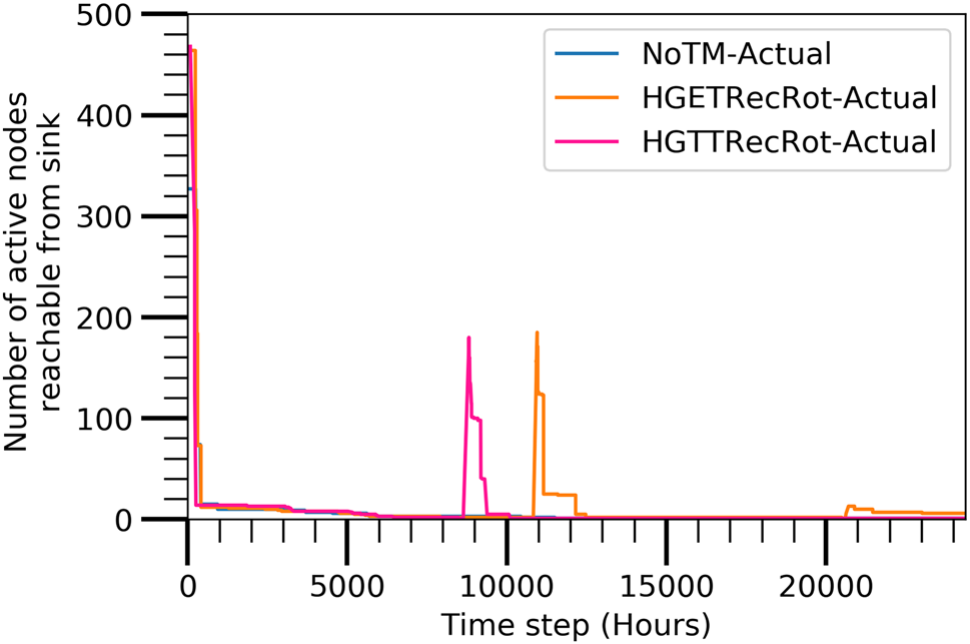
Actual evaluated active nodes reachable from sink.

**Figure 9:**
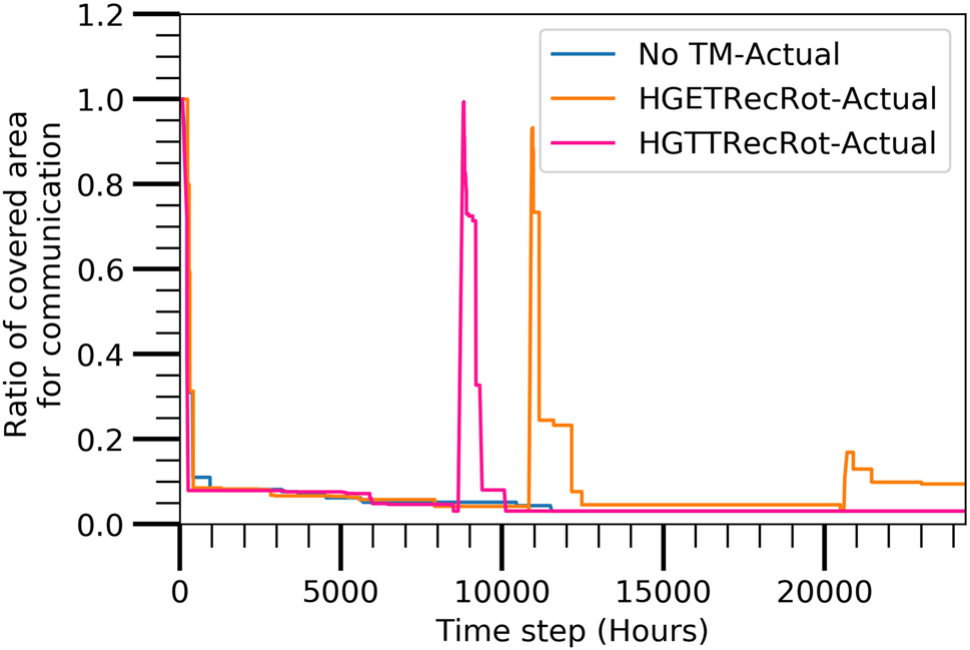
Actual evaluated covered communication area.

**Figure 10:**
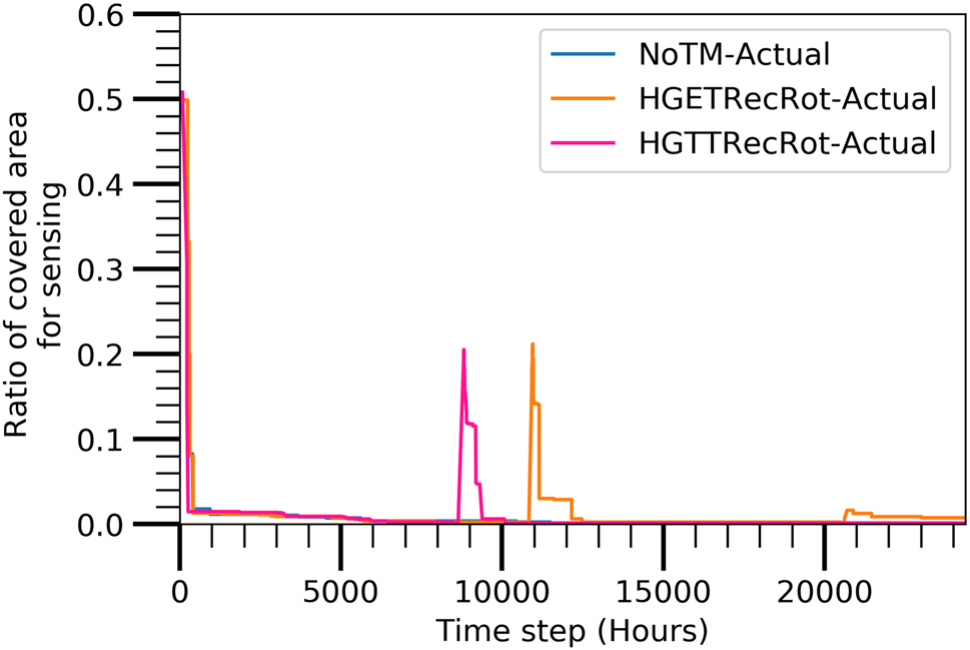
Actual evaluated covered sensing area.

Figure 7 illustrates the number of utilized nodes in the network after applying the HGTTRecRot, HGETRecRot TM and NoTM with A3Cov TC algorithms. The figure reveals that the NoTM option requires few nodes less with respect to the HGTTRecRot and HGETRecRot but is unable to furnish an elongated lifespan of the network which is seen in Figs. 8, 9, and 10. The HGTTRecRot and HGETRecRot preserve the better connectivity coverage of the network over the lifetime as found in the figures.

#### Active nodes reachable from sink

The number of active nodes for applying the three algorithms which can be connected from sink node following remaining energy of the nodes at various time steps is shown in Fig. 8. The most significant facts are seen at the time steps of 8796 and 10920 where the number of active nodes are increased considerably in the case of HGTTRecRot and HGETRecRot, respectively. This behavior is due to the controlling mechanism of the maintenance policy which also enables the network with reduced nodes to provide better network connectivity in the critical phase. Therefore, it is clear that the maintenance techniques are working perfectly in our network design. A small increment is also seen at time step 20,705 for only HGETRecRot algorithm. However, there is no increments for NoTM algorithm like the other two algorithms. It is clear that the HGETRecRot achieves a longer lifetime of the network in comparison to the other two algorithms.

#### Communication coverage area

Figure 9 shows the ratio of the covered communication area with respect to the target area of the network at the different time steps for the above three algorithms. All the three algorithms cover approximately entire area initially but they are decreased afterwards. The HGTTRecRot and HGTTRecRot algorithms again cover entire area approximately at the time steps of 8796 and 10,920, respectively and the decremented trends are again seen afterwards. This increments are resulted also from the maintenance process of the corresponding algorithms. Finally, a small increment from only the HGETRecRot is found at the time step of 20,705. This findings also indicates that the HGETRecRot has long lasting lifetime of the network reserving the entire area more connective than others.

#### Sensing coverage

Figure 10 displays the ratio of the covered sensing areas with respect to the target area at different time steps for the three algorithms. The sensing coverage area has similar trend like the communication coverage area and active nodes reachable from sink node. It similarly shows that the HGETRecRot has the most durable lifetime of the network reserving a larger sensing coverage area than the other algorithms.

### Prediction model’s performance evaluation

**Figure 11:**
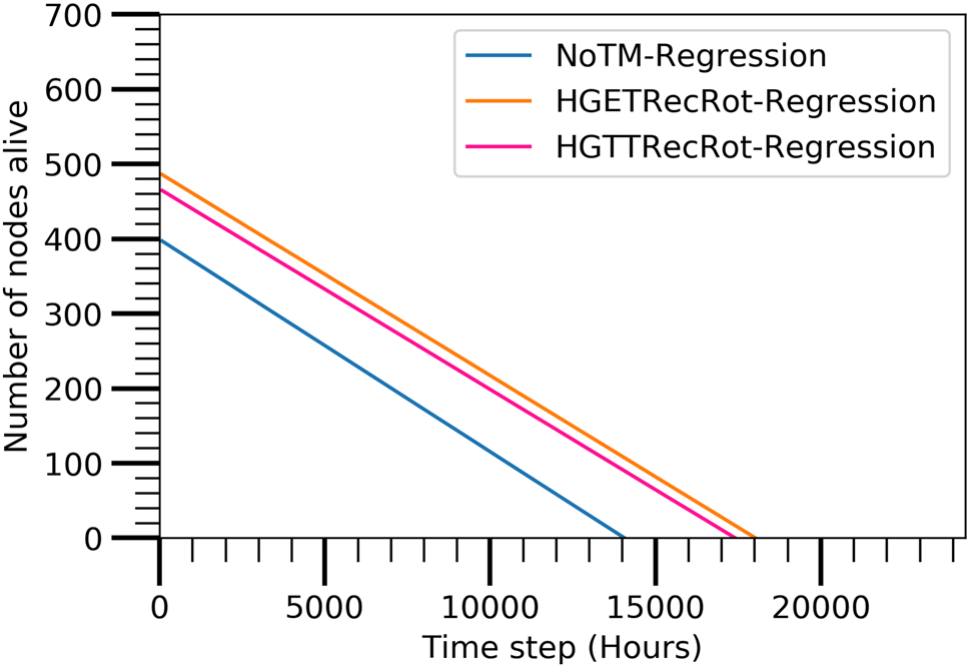
Alive node based on prediction model.

**Figure 12:**
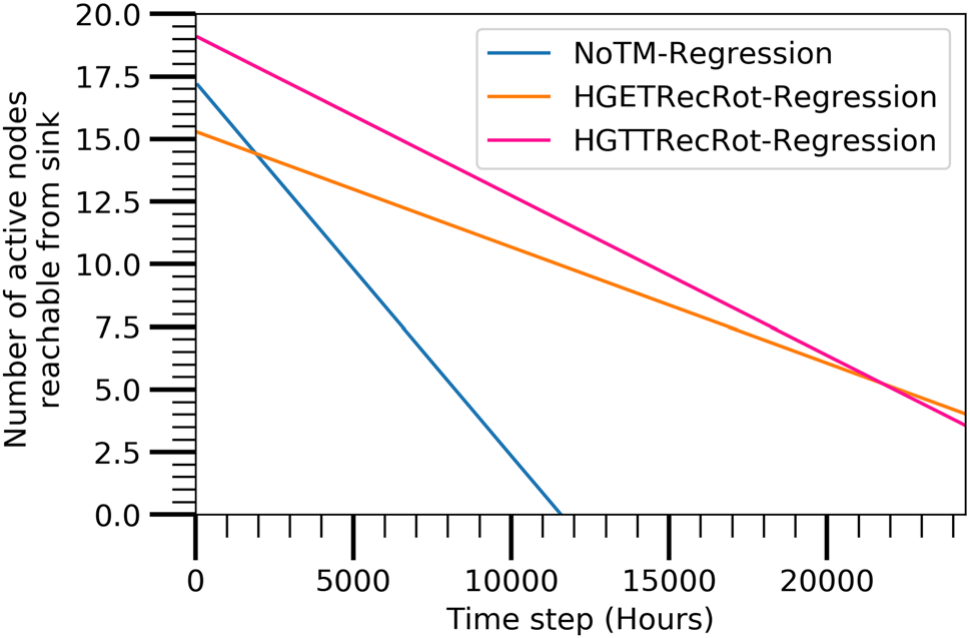
Active nodes reachable from sink based on prediction model.

**Figure 13:**
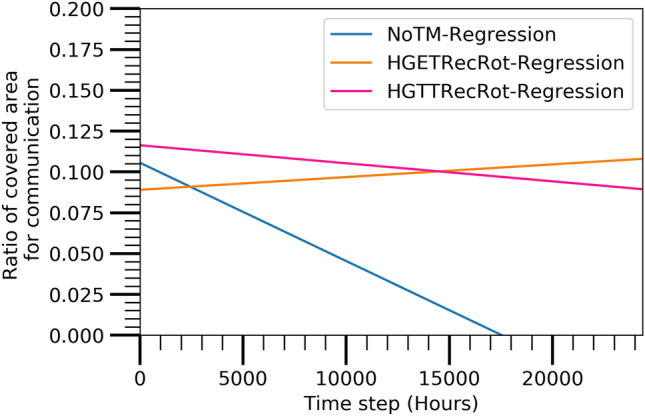
Covered communication area based on prediction model.

**Figure 14:**
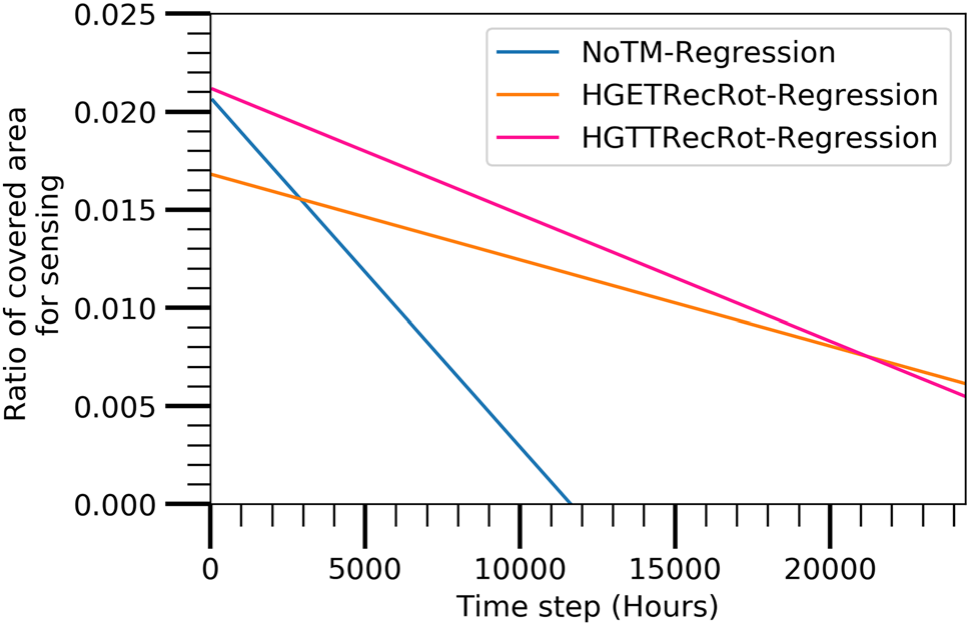
Covered sensing area based on prediction model.

The performance of the prediction model is discussed in this section to identify the best combination of TM and TC algorithm. Figures 11,  12,  13, and  14 show the best combination for analyzing the collected data from the Atarraya simulation software for the number of alive nodes, the number of active nodes reachable from sink, the ratio of covered communication area, and the ratio of covered sensing area, respectively at the different time steps for the three algorithms. The figures are generated from the linear regression analysis. It is observed in Fig. 11 that the HGTTRecRot and HGETRecRot algorithms yield approximately the similar better utilizing the nodes in the network than the NoTM algorithm.

Figure 12 shows that the HGTTRecRot has higher active nodes initially than HGETRecRot and NoTM algorithms but the HGETRecRot does better than others during the ending time steps. Similarly, the Figs. 13 and 14 show resembling trend as in Fig. 12. Therefore, it can be concluded that HGETRecRot approach has better lifespan than the other two algorithms.

#### Coefficients of the model

As pointed out in section “Prediction model”, the coefficients of the linear regression model, namely, intercept, and slope represent the intersection between the linear fitted line and the Y-axis, and the slope of the linear fitted line, respectively. Moreover, linear regression model fits the linear line using the coefficients so that the squared residual sum between observed targets of dataset and the targets predicted by linear approximation is minimum. These regression coefficients leave a numerical interpretation of the behaviours of the model. The regression coefficients of the prediction model for the different cases using related terminology described in that section are summarized in Table 3.

**Table 3:** The values of regression coefficients, CoD, and RMSE obtained from the prediction model.

Case	Algorithms	$\varvec{\beta _0}$	$\varvec{\beta _1}$	CoD	RMSE
Alive node	NoTM	399.2541	− 2.84e–2	0.8352	53.3215
	HGETRecRot	487.9034	− 2.71e–2	0.8908	54.1447
	HGTTRecRot	466.6844	− 2.68e–2	0.8394	63.0591
Active nodes reachable from sink	NoTM	17.2794	− 1.49e–3	0.0676	23.4237
	HGETRecRot	15.3037	− 4.63e–4	0.0073	30.8354
	HGTTRecRot	19.1246	− 6.38e–4	0.0094	35.1484
Covered communication area	NoTM	0.1056	− 6.02e–6	0.1426	0.0624
	HGETRecRot	0.0890	7.78e–7	0.0014	0.1177
	HGTTRecRot	0.1163	− 1.10e–6	0.0011	0.1753
Covered sensing area	NoTM	0.0207	− 1.18e–6	0.0601	0.0297
	HGETRecRot	0.0168	− 4.38e–7	0.0054	0.0339
	HGTTRecRot	0.0212	− 6.45e–7	0.0076	0.0396

#### Coefficient of determination

These values of CoD of three prediction models, NoTM, HGETRecRot, and HGTTRecRot with A3Cov, in different cases are listed in Table 3. The values of coefficient of determination (CoD) for NoTM, HGETRecRot, and HGTTRecRot with A3Cov in the case of alive nodes are obtained 0.8352, 0.8908, and 0.8394, respectively. Since higher value of CoD represents the less probability of variation in prediction, hence, HGETRecRot has better value of CoD than NoTM and HGTTRecRot algorithms. Thus HGETRecRot algorithm offers better confidence in predictions. The values of CoD for NoTM, HGETRecRot, and HGTTRecRot with A3Cov are obtained 0.0676, 0.0073, and 0.0094, respectively, in the case of active nodes reachable from sink. Therefore, it is clear that NoTM provides the better CoD than the other two algorithms. The values of CoD for NoTM, HGETRecRot, and HGTTRecRot with A3Cov are obtained 0.1426, 0.0014, and 0.0011, respectively, in the case of covered communication area. In this case, the NoTM algorithm also has better CoD value than the other two algorithms. The values of CoD for NoTM, HGETRecRot, and HGTTRecRot with A3Cov in the case of covered sensing area are obtained 0.0601, 0.0054, and 0.0076, respectively. The NoTM algorithm also has better CoD in this case than others. An important insight about the CoD values is that the values in the cases of active node from sink, covered communication area, and covered sensing area are not higher as in the case of alive nodes. This is because of the enormous improvement of the performances at time steps 8796 for the HGTTRecRot with A3Cov and 10920 and 20705 for the HGETRecRot with A3Cov. Therefore, this insight suggests to identify the optimized best deployment using different algorithms on the existing data set rather than estimating the future value.

#### Root mean squared error

In the case of alive nodes, the values of root mean squared error (RMSE) for the NoTM, HGETRecRot, and HGTTRecRot with A3Cov are obtained 53.3215, 54.1447, and 63.0591, respectively. In the case of active nodes reachable from sink, RMSE values for the NoTM, HGETRecRot, and HGTTRecRot with A3Cov are obtained 23.4237, 30.8354, and 35.1484, respectively. In the case of covered communication area, the RMSE values are obtained 0.0624, 0.1177, and 0.1753, respectively, for the NoTM, HGETRecRot, and HGTTRecRot with A3Cov, respectively. In the case of covered sensing area, 0.0297, 0.0339, and 0.0396, RMSE values are achieved for the NoTM, HGETRecRot, and HGTTRecRot, respectively with A3Cov TC protocol. Table 3 presents the estimated RMSE values of all three algorithms in different cases. The lower RMSE value represents less variation and better confidence in the prediction. Hence, it is clear that the NoTM has better RMSE than the other two algorithms in all of the cases. And, the HGETRecRot has better RMSE value than the HGTTRecRot.

## Conclusion

We propose a hybrid approach to enhance the lifetime of WSNs, explicitly targetting the nuclear power plant monitoring applications. We consider the $1014 \, {\text{m}} \times 1014 \, {\text{m}}$ area of a nuclear power plant, i. e., the Rooppur Nuclear Power Plant scenario in Bangladesh, in our application for the uniformly distributed optimum nodes deployment. We evaluate the network’s performance regarding four performance metrics: the number of total alive nodes, the number of total active nodes connected from the sink, the total covered communication area, and the total covered sensing area. Our research outcomes present several approaches to achieve an extended lifespan, better communication connectivity, and better-covered sensing coverage of WSNs. However, we observe that the HGETRecRot algorithm increases the lifetime of the WSN in most cases compared to HGTTRecRot and NoTM algorithms. In future work, we can deploy different moving sensor nodes, analyze the time and energy-based sensitivities of the WSNs, and apply deep learning techniques to the prediction model.
